# High-Frequency Global Postoperative Status PROMs Track Pain Peaks and Analgesic Use After Degenerative Lumbar Spine Surgery

**DOI:** 10.1177/21925682261449153

**Published:** 2026-06-05

**Authors:** Pavlina Lenga, Robin Fleige, Max Christian Blumenstock, Matthias Ganzinger, Sebastian Ille, Sandro M. Krieg, Martin Dugas

**Affiliations:** 1 Department of Neurosurgery, 9144 Heidelberg University Hospital, Heidelberg, Germany; 2 Institute of Medical Informatics, 9144 Heidelberg University Hospital, Heidelberg, Germany

**Keywords:** lumbar spine surgery, patient-reported outcome measures, postoperative pain, digital health, recovery trajectories

## Abstract

**Study Design:**

Prospective observational cohort study.

**Objective:**

Standard postoperative follow-up often fails to capture rapid fluctuations in symptoms after degenerative lumbar spine surgery. This study evaluated the construct validity of a high-frequency, low-burden “Global Postoperative Status” item as an exploratory measure of concurrent early post-discharge recovery burden.

**Methods:**

Prospective observational cohort study of 257 patients undergoing surgery for lumbar disc herniation or spinal canal stenosis who contributed 1627 postoperative status assessments over 6 weeks via secure mobile surveys every 2-3 days. Construct validity was examined through associations with pain-peak frequency, pain-peak intensity (0-10), analgesic use, and wound pain. Because peak intensity was conditionally collected only when pain peaks were reported, the primary mixed-effects complete-case model included 209/1627 assessments (82 subjects); broader sensitivity analyses used larger available-case datasets.

**Results:**

Worse Global Status scores were consistently associated with greater concurrent pain burden. Median status was 60 (IQR 50-80) when no pain peaks were reported vs 40 (IQR 20-50) when pain peaks occurred >5/day. Mean status was 67.2 without analgesics and 41.9 with combined opioid plus non-opioid analgesics. Status correlated inversely with pain peak intensity (Spearman rho = −0.53, *P* < 0.001). In the exploratory complete-case mixed-effects model, each 1-point increase in pain peak intensity was associated with a 4.53-point lower status score (95% CI -6.09 to −2.97; *P* < 0.001); this direction was preserved in broader sensitivity analyses that retained more observations.

**Conclusions:**

The high-frequency Global Status item showed exploratory construct-validity evidence as a low-burden indicator of concurrent postoperative pain burden and analgesic use after lumbar spine surgery. The findings do not establish responsiveness, test-retest reliability, criterion validity against established PROMs, or decision-support utility, and prospective validation studies are required before clinical implementation claims can be made.

## Introduction

Recovery following degenerative spine surgery is heterogeneous, and early postoperative pain is an important component of patient experience and short-term recovery. Prior observational work suggests that poor early pain control may be associated with less favorable later patient-reported outcomes, but routine follow-up still samples this period only sparsely.^1^ As a result, the interval between discharge and the next scheduled clinic visit often remains a practical post-discharge “black box.” Conventional PROM collection may miss short-lived symptom excursions, and retrospective reporting is vulnerable to recall distortion: within-subject diary research shows that recalled daily pain is disproportionately influenced by peak intensity and end-of-day symptoms (the “peak-end” heuristic) rather than by the full time-weighted experience.^2^ High-frequency assessment therefore offers a way to describe symptom fluctuations more directly, but it should not be assumed to capture a uniquely “true” recovery trajectory in the absence of broader validation.

Mobile health approaches and ecological momentary assessment (EMA) offer a feasible way to obtain brief, repeated, real-world measurements. Recent lumbar spine work has shown that momentary EMA measures can converge with established PROM frameworks while capturing symptom dynamics more granularly than cross-sectional instruments, and the broader neurosurgical literature describes expanding applications of mobile monitoring for perioperative assessment.^3^ Electronic symptom capture has also been shown to be comparable to conventional collection methods in clinical pain studies.^4^ However, feasibility of frequent measurement does not, by itself, establish that a single-item global score is fully validated or interchangeable with established multidomain instruments.^5^

Importantly, brevity can improve feasibility when repeated sampling is required, but it also has trade-offs. Single-item global PROMs can provide useful summary signals and in some settings have shown acceptable validity, reliability, and responsiveness compared with longer questionnaires. Spine-specific work on the Subjective Spine Value likewise suggests that a one-item measure can correlate with established outcomes. At the same time, single-item measures cannot disentangle specific domains such as pain intensity, disability, sleep, mood, or wound-related symptoms and may show different measurement properties across clinical contexts. They therefore require targeted validation for the intended use case rather than being assumed to replace instruments such as ODI or PROMIS.^6,7^

In this study, we evaluated the construct validity of a high-frequency, low-burden global postoperative status PROM (scale 1-100) as an exploratory measure to characterize the early post-discharge period following lumbar spine decompression surgery for spinal canal stenosis or lumbar disc herniation. We hypothesized a priori that lower status scores would be associated with more frequent and more intense pain peaks, greater analgesic use, and higher wound pain across repeated postoperative assessments. We did not aim to establish criterion validity against established multidomain PROMs, responsiveness, test-retest reliability, predictive performance, or clinical decision thresholds. Exploratory subgroup analyses examined whether these directional associations were similar across age and sex.

## Methods

### Study Design and Ethical Oversight

This study is a secondary analysis of prospectively collected electronic patient-reported outcome (ePROM) data, integrated into the routine clinical workflow at the neurosurgical outpatient clinic of Heidelberg University Hospital (UKHD). All procedures adhered to the Declaration of Helsinki. The clinical implementation of the institutional ePROM platform (MyEDC) was approved by the local ethics committee (S607/2023). The secondary analysis of this routinely collected registry data was authorized under the institutional neurosurgical registry approval (S096/2025), which waived the requirement for additional written consent. Because the high-frequency ePROM pathway was embedded in routine care rather than designed as a formal validation trial, recruitment/refusal logs and structured reasons for non-completion were not prospectively captured in the registry extract.

## Participants and Surgical Procedures

We enrolled consecutive patients with degenerative lumbar spine pathology who underwent surgery for lumbar disc herniation (LDH) or spinal canal stenosis (SCS). Following surgery, patients entered the high-frequency ePROM pathway as part of standard postoperative care. Surgical indications were confirmed via multidisciplinary review based on concordant clinical symptoms and neuroimaging (MRI/CT). All procedures were performed by board-certified neurosurgeons according to institutional standards, specifically microsurgical discectomy via interlaminar fenestration for LDH and decompression via interlaminar fenestration with targeted undercutting for SCS. Patients were eligible for analysis if they entered the routine postoperative ePROM pathway and contributed at least 1 valid postoperative Global Status assessment. Patients who did not complete any valid postoperative status entry could not contribute to the analytic datasets; this design feature may preferentially exclude less engaged patients as well as some patients with low or high symptom burden and therefore introduces potential selection bias. No additional a priori exclusion criteria were applied within this registry-based cohort. Because this was an exploratory secondary analysis of routinely collected consecutive cases, no formal sample size calculation was performed; all eligible patients available in the registry extract were included. This yielded 257 patients for the primary outcome analysis.

### Digital Platform and Data Collection

Data were captured using MyEDC, a browser-based web application accessible on smartphones, tablets, and computers through personalized email links. Assessments were scheduled approximately every 2-3 days for up to 6 weeks within the routine digital follow-up workflow. For longitudinal analyses, RepeatKey was treated as an ordinal index of assessment order rather than as an exact elapsed-time variable. We chose this approach because the present study focused on directionality of repeated within-patient symptom tracking, whereas actual survey spacing in routine care showed modest deviations around the intended 2-3-day cadence due to weekends, workflow, and variable patient adherence. Using an ordinal sequence reduced the influence of these small calendar irregularities when summarizing early trajectories, but it does not provide a precise time-scale analysis. Source-system timestamps were retained and are reported descriptively; structured reasons for missed or delayed responses were not prospectively captured. In the primary outcome dataset, the 257 included patients contributed 1627 valid Global Status assessments, corresponding to a median of 4 assessments per patient (IQR 3-7; mean 6.3; range 1-43). No minimum completion requirement beyond 1 valid postoperative Global Status entry was imposed; each analysis used all available observations meeting the variable-specific data requirements (Table 1).

**Table 1.** Data Availability and Sample Sizes (Subjects and Assessments)

Characteristic	Value
Panel a. Subject-level cohort characteristics (n = 257)	
Age, mean +/- SD, years	64.3 +/- 20.5
Female	130/257 (50.6%)
Male	127/257 (49.4%)
Valid global status assessments per patient, median (IQR)	4 (3-7)
Valid global status assessments per patient, mean (range)	6.3 (1-43)
Panel B. Assessment-level data availability for main analyses	
Overall postoperative status	1627 assessments from 257 subjects
Pain peak frequency	551 assessments from 156 subjects
Pain peak intensity	217 assessments from 87 subjects
Analgesic use	433 assessments from 135 subjects
Wound pain	1604 assessments from 255 subjects
Complete-case mixed-effects model dataset	209 assessments from 82 subjects
Complete-case wound-pain model dataset	202 assessments from 81 subjects

Characteristic	Value
Panel A. Subject-level cohort characteristics (n = 257)	
Age, median [IQR], years	63 [54-72]
Female	130/257 (50.6%)
Male	127/257 (49.4%)
Valid global status assessments per patient, median (IQR)	4 (3-7)
Valid global status assessments per patient, mean (range)	6.3 (1-43)
Panel B. Assessment-level data availability for main analyses	
Overall postoperative status	1627 assessments from 257 subjects
Pain peak frequency	551 assessments from 156 subjects
Pain peak intensity	217 assessments from 87 subjects
Analgesic use	433 assessments from 135 subjects
Wound pain	1604 assessments from 255 subjects
Complete-case mixed-effects model dataset	209 assessments from 82 subjects
Complete-case wound-pain model dataset	202 assessments from 81 subjects

### Outcome Measures

#### Primary Outcome

Global Status: Overall Postoperative Status (1-100): A single-item visual analogue scale asking patients to rate how they felt overall “today” after surgery. The item was numerically anchored from 1 (worst postoperative status) to 100 (best postoperative status), with higher scores indicating better status.^1^ This locally implemented item had not been formally validated previously as a standalone postoperative spine PROM; the present study therefore evaluated its construct validity against concurrent pain-related measures. Because established multidomain instruments such as ODI or PROMIS were not administered at the same high-frequency cadence in this routine-care pathway, direct criterion/convergent comparisons with those measures were not possible in the present analysis (Table 2).

**Table 2.** Descriptive Statistics for Overall Postoperative Status

(A) Overall Postoperative Status (1-100)								
Assessments (n)	Subjects (n)	Mean	SD	Median	Q1	Q3	Min	Max
1627	257	64.2	25.48	70	50	85	1	100

(B) Overall Status Stratified by Pain Peak Frequency						
Pain peak frequency	n	Median	q1	q3	Mean	sd
None	351	60	50	80	60.27	22.26
1-2/day	108	60	40	75	56.2	24.18
3-5/day	50	40	30	64	44.22	22.06
>5/day	33	40	20	50	34.64	21.95

(C) Overall Status Stratified by Analgesic Category						
Analgesic use	n	Median	q1	q3	Mean	sd
No analgesics	184	70	53.75	85	67.17	22.6
Opioid analgesics	38	50	20	71.25	47.68	29.16
Non-opioid analgesics	188	50	40	65	50.74	21.6
Opioid + non-opioid	16	40	25.75	67	41.94	27.15

### Pain Volatility (Peak Measures)

#### Pain Peak Frequency

“Pain peaks” referred to patient-reported transient postoperative exacerbations of pain during the day (“Schmerzspitzen im Tagesverlauf”). Patients categorized how often such peaks occurred (None; 1-2 times/day; 3-5 times/day; >5/day).^2^ This item captures a patient-perceived symptom construct rather than a standardized physiologic endpoint and may therefore vary in interpretation across individuals.

#### Pain Peak Intensity (0-10)

If peaks were present, patients rated the maximum intensity of those peaks on a numeric rating scale from 0 to 10.^3^ This item was conditionally displayed only when peaks were reported; therefore, missingness is structural for assessments with peak frequency = None.

### Pain Management and Wound Pain

• Analgesic Use: Categorized into 4 levels: No analgesics, Non-opioid analgesics, Opioid analgesics, and Combined (Opioid + Non-opioid).^4^
• Wound Pain (0-10): An NRS assessing current wound site pain.^5^

### Demographics

Age was derived from the electronic record. Sex was inferred from the completion of sex-specific questionnaire modules (female vs male); subjects with conflicting or missing module data were classified as ambiguous or unknown, respectively, and included only in exploratory robustness checks.

### Data Preprocessing

Response values were range-checked and converted to numeric formats where applicable. Admissible ranges were prespecified as follows: Global Status 1-100, wound pain 0-10, pain peak intensity 0-10, pain peak frequency 1-4, and analgesic category 1-4. Across these core analytic variables, 23 of 4455 non-missing raw entries (0.52%) were outside the admissible range and were recoded as missing: 20/1624 wound-pain entries (1.23%) and 3/220 pain-peak-intensity entries (1.36%). No out-of-range values occurred for Global Status, pain peak frequency, or analgesic category. Analyses were then performed on variable-specific available-case or complete-case datasets as appropriate. To account for the longitudinal design, we used the sequential assessment index (RepeatKey)^6^ rather than exact elapsed time. For early trajectory analyses, we focused on RepeatKey 1-6 because observations became increasingly sparse at later indices; these summaries are therefore descriptive, reflect assessment order rather than exact postoperative day, and should be interpreted with the concurrent attrition pattern in mind.

### Statistical Analysis

All analyses were conducted using R statistical software (version 4.2.2, R Foundation for Statistical Computing, Vienna, Austria); figures were generated with matplotlib. Continuous variables are reported as mean ± SD or median (IQR) and categorical variables as n (%). Construct validity of the overall postoperative status item was assessed using prespecified concurrent pain-related comparators: pain peak intensity, pain peak frequency, analgesic use, and wound pain. Associations were summarized with Spearman correlations (and within-subject centered correlations to reduce between-subject confounding), Kruskal–Wallis tests across peak-frequency and analgesic categories, and mixed-effects models with a random intercept per subject. Because pain peak intensity was conditionally asked only when peaks were reported, the primary mixed-effects model necessarily relied on complete cases and therefore should be interpreted as an exploratory association model rather than a definitive multivariable validation model. Sensitivity analyses included restriction to assessments with peaks present, a simplified 2 × 2 grouping (any peaks × any analgesics) using subject-clustered robust standard errors, and an extension model including wound pain. Early recovery trends across RepeatKey 1-6 were evaluated using cluster-robust linear regression (status) and generalized estimating equations (GEE; binary outcomes: any peaks/any analgesics) with binomial family, logit link, exchangeable working correlation, and subject-level clustering; odds ratios per +1 assessment order with 95% confidence intervals were reported. Statistical significance was defined as a two-sided *P*-value <0.05 without multiplicity adjustment because analyses were designed to test convergent construct-validity patterns across related prespecified domains; individual *P*-values are therefore interpreted descriptively. To better characterize missingness, we summarized completeness by assessment order, compared observed subject-level characteristics between subjects included and excluded from the complete-case model, and compared concurrent status and wound-pain distributions between complete and incomplete assessments. These comparisons suggested a mixture of structural missingness and informative non-response rather than clearly missing-completely-at-random data. Formal multiple imputation across all assessments was not pursued because peak-intensity values are structurally undefined when no pain peaks are reported and because the available auxiliary variables were limited for defensible MAR modeling; instead, robustness was examined through broader sensitivity models that omitted peak intensity and retained more observations. Because the study was not designed for causal or predictive inference, models were not interpreted as estimating treatment effects or clinical thresholds, and limited registry capture prevented comprehensive adjustment for potential confounders such as surgical complexity, baseline function, and comorbidities. All inferential statistics and tabular outputs were generated from R. Python/matplotlib was used only for figure rendering from analysis-ready summaries exported from R. For descriptive sample characterization only, we additionally summarized available baseline anthropometric fields and derived an exploratory percentage disability summary from the structured 10-item baseline disability block by recoding 1-6 item responses to 0-5 and averaging across available items when at least 8/10 items were present; this derived summary was not used in the primary outcome models. All table values and extremely small *P*-values were re-checked against the original R output and are reported in scientific notation where appropriate. To streamline presentation, the complementary correlation-based and groupwise association results are summarized together in Table 3 rather than repeated in multiple sections. Where vertical error bars are shown in Figures 2 and 3, they represent 95% confidence intervals.

**Table 3.** Bivariate Associations

(A). Bivariate Associations Between Overall Status and Pain Burden Variables					
Association or comparison	Method/Test	Estimate	*P*-value	Assessments (n)	Subjects (n)
Overall status vs pain peak intensity	Spearman	rho = −0.530	8.05e-17	213	85
Overall status vs pain peak intensity	Pearson	r = −0.524	1.89e-16	213	85
Overall status vs pain peak intensity (within-subject centered)	Pearson	r = −0.386	5.7e-09	213	85
Overall status vs pain peak frequency	Spearman	rho = −0.249	4.1e-09	542	156
Pain peak frequency vs pain peak intensity	Spearman	rho = 0.595	4.34e-22	216	86
Status by pain peak frequency	Kruskal-wallis	H = 48.69	1.52e-10	542	156
Status by analgesic category	Kruskal-wallis	H = 61.92	2.28e-13	426	135

Association or comparison	Method/Test	Estimate	*P*-value	Assessments (n)	Subjects (n)
Overall status vs pain peak intensity	Spearman	rho = -0.530	8.05e-17	213	85
Overall status vs pain peak intensity	Pearson	r = -0.524	1.89e-16	213	85
Overall status vs pain peak intensity (within-subject centered)	Pearson	r = -0.386	5.7e-09	213	85
Overall status vs pain peak frequency	Spearman	rho = -0.249	4.1e-09	542	156
Pain peak frequency vs pain peak intensity	Spearman	rho = 0.595	4.34e-22	216	86
Status by pain peak frequency	Kruskal-Wallis	H = 48.69	1.52e-10	542	156
Status by analgesic category	Kruskal-Wallis	H = 61.92	2.28e-13	426	135

(B) Kruskal–Wallis Tests for Group Differences in Overall Status		
Test	H	*P*-value
Kruskal–Wallis: status by pain peak frequency	48.69	1.52e-10
Kruskal–Wallis: status by analgesic category	61.92	2.28e-13

## Results

### Study Cohort and Data Availability

The study cohort comprised 257 patients with a mean age of 64.3 +/- 20.5 years.^7^ These patients contributed 1627 assessments with available overall postoperative status data (1-100; higher = better), corresponding to a median of 4 assessments per patient (IQR 3-7; mean 6.3; range 1-43) (Table 1). The cohort exhibited a balanced sex distribution (female: n = 130 [50.6%]; male: n = 127 [49.4%]).^8^ Subject-level cohort characteristics and assessment-level data availability for the main analytic variables are summarized separately in Table 1. Pain peak frequency was available in 551 assessments from 156 subjects, pain peak intensity in 217 assessments from 87 subjects, analgesic use in 433 assessments from 135 subjects, and wound pain in 1604 assessments from 255 subjects. The complete-case dataset for the mixed-effects model comprised 209 assessments from 82 subjects. Because the primary mixed-effects model therefore used only 12.8% of all status assessments, its estimates require cautious interpretation. Observed subject-level characteristics of the 82 subjects included in the primary complete-case model were similar to those of the 175 excluded subjects with respect to age, sex, earliest postoperative status, and earliest wound pain (Table S9). However, these observed similarities do not exclude selection on unmeasured factors or assessment-level symptom state.

### Cross-Sectional Distribution and Bivariate Associations

**Figure 1.**
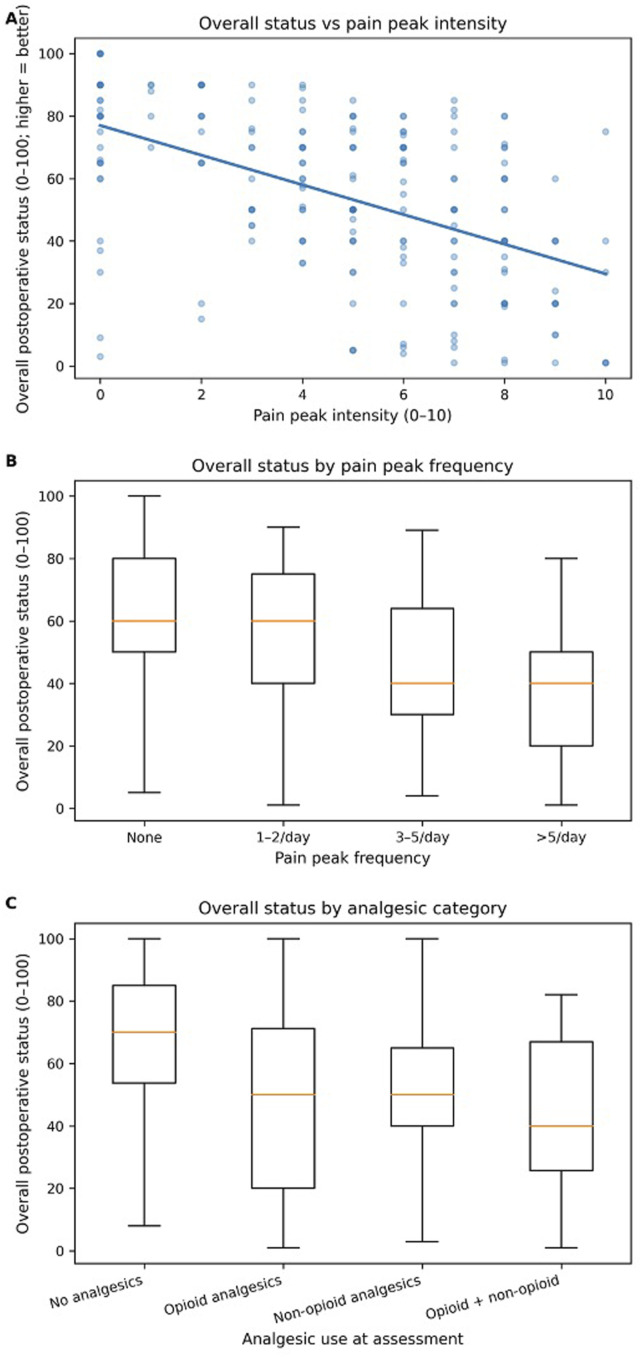
93x195mm (144 x 144 DPI)

Overall postoperative status exhibited substantial heterogeneity across assessments (mean 64.2, SD 25.5; median 70, IQR 50-85; range 1-100) (Table 2A). Status was lower at assessments reporting greater pain burden. In pairwise comparisons, assessments reporting no pain peaks showed higher status than those reporting very frequent peaks (>5/day) (median 60 [IQR 50-80] vs 40 [IQR 20-50]; *P* = 5.9 × 10^-9) (Table 2B). Similarly, status was higher when no analgesics were reported than when opioid-containing regimens were used, both for opioid-only use (median 70 [IQR 53.8-85] vs 50 [IQR 20-71.3]; *P* = 7.2 × 10^-5) and combined opioid/non-opioid use (median 70 [IQR 53.8-85] vs 40 [IQR 25.8-67]; *P* = 3.6 × 10^-4) (Figure 1, Table 2C). Correlation and nonparametric association analyses are summarized together in Table 3A. Overall status was strongly and inversely associated with pain peak intensity (Spearman rho = −0.530, *P* = 8.05 × 10^-17, 213 assessments), and this association persisted after within-subject centering (Pearson r = −0.386, *P* = 5.70 × 10^-9). Status also showed an inverse association with pain peak frequency (Spearman rho = −0.249, *P* = 4.1 × 10^-9, 542 assessments), while pain peak frequency correlated positively with pain peak intensity (Spearman rho = 0.595, *P* = 4.34 × 10^-22, 216 assessments). Complementary groupwise tests confirmed that status differed across pain peak frequency categories (Kruskal-Wallis H = 48.69, *P* = 1.52 × 10^-10) and across analgesic categories (Kruskal-Wallis H = 61.92, *P* = 2.28 × 10^-13) (Table 3B).

### Patterns Across Repeated Assessments

**Figure 2.**
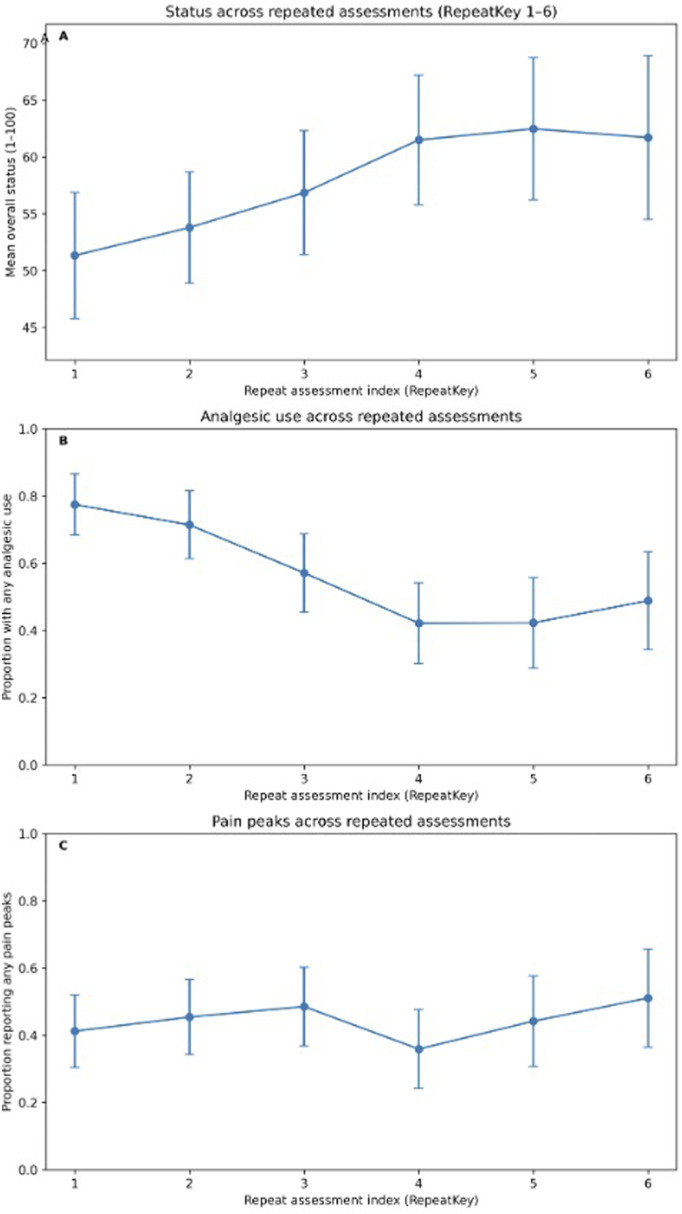
83x149mm (144 x 144 DPI)

**Figure 3.**
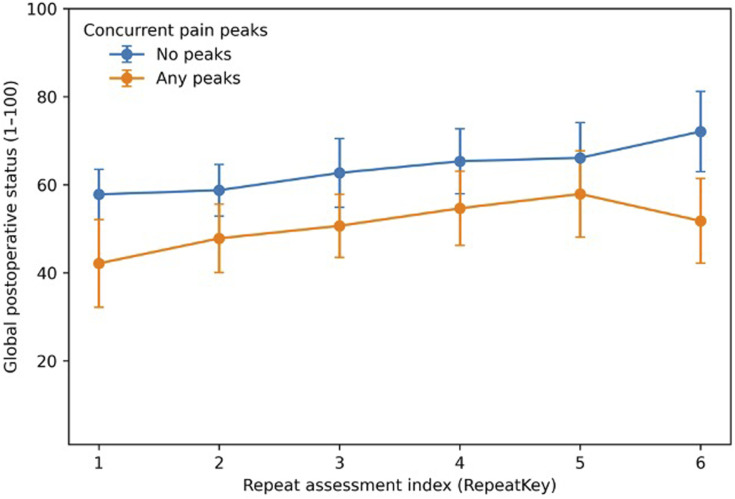
113x77mm (144 x 144 DPI)

Figure 2 and Table 4 summarize available assessments at RepeatKey 1-6 rather than restricting analysis to patients with complete six-assessment follow-up; accordingly, the number of contributing subjects decreased from 80 at RepeatKey 1 to 45 at RepeatKey 6. These summaries reflect assessment order rather than exact postoperative day and are descriptive because later follow-up was progressively less complete. Across these repeated assessments, mean global postoperative status increased from 51.3 to 61.7, while the proportion reporting any analgesic use declined from 77.5% to 48.9% (Table 4A; Figure 2A–B). By contrast, the proportion reporting any pain peaks remained within a relatively narrow range across RepeatKey 1-6 (35.9%–51.1%) (Table 4B; Figure 2C). Taken together, these descriptive trends indicate improving overall recovery and declining medication requirements, whereas intermittent breakthrough pain remained common and did not decline in parallel. In a cluster-robust regression restricted to early repeated assessments, global postoperative status nevertheless remained significantly lower when pain peaks were reported at the same assessment (beta = −15.45, *P* = 0.000426), despite the overall upward trend over time (Figure 3). This corresponded to relative coverage of 100.0%, 96.3%, 87.5%, 80.0%, 65.0%, and 56.3% at RepeatKey 1-6, respectively, equivalent to a 43.8% reduction in available assessments by RepeatKey 6. Because structured reasons for non-completion were not prospectively recorded in this routine-care registry, these values should be interpreted as descriptive implementation coverage rather than adjudicated dropout categories, and temporal patterns may partly reflect informative attrition.

**Table 4.** Mixed-Effects Model (Random Intercept per Subject): Predictors of Overall Postoperative Status

(A) Model Metadata					
Model	Outcome	Dataset	Assessments (n)	Subjects (n)	ICC (random intercept)
Mixed-effects linear model (random intercept per subject)	Overall postoperative status (1-100)	Complete cases: status + peak frequency + peak intensity + analgesics	209	82	0.218

(B) Fixed-Effect Estimates (Reference Categories: Pain Peak Frequency = None; Analgesic Use = No Analgesics)					
Term	Coef	SE	CI low	CI high	P-value
Intercept	72.661	3.988	64.845	80.477	3.56e-74
Pain peak frequency: 1-2/day vs none	8.187	5.099	−1.807	18.182	0.108
Pain peak frequency: 3-5/day vs none	1.638	5.968	−10.059	13.336	0.784
Pain peak frequency: >5/day vs none	−8.336	7.635	−23.301	6.629	0.275
Analgesics: Opioid vs none	0.522	6.219	−11.668	12.711	0.933
Analgesics: Non-opioid vs none	−0.092	3.85	−7.638	7.454	0.981
Analgesics: Opioid + non-opioid vs none	9.447	7.646	−5.54	24.434	0.217
Pain peak intensity (per +1 on 0-10 scale)	−4.528	0.796	−6.089	−2.968	1.29e-08

### Multivariable Models

To account for repeated measures nested within subjects, we fitted a random-intercept linear mixed-effects model using the exploratory complete-case dataset (209 assessments from 82 subjects; ICC = 0.218) (Table 5). In this model, pain peak intensity remained independently associated with worse overall status (β −4.53 points per +1 on the 0-10 scale; 95% CI -6.09 to −2.97; *P* = 1.29 × 10^-8) (Table 5). After adjustment for peak intensity, neither pain peak frequency nor analgesic category showed statistically significant independent associations. Given the restricted sample and non-random completeness pattern, these coefficients should be interpreted as supportive construct-validity associations rather than definitive effect estimates. In an extension mixed-effects model that additionally included wound pain (202 assessments from 81 subjects), wound pain remained independently associated with worse status (beta −2.85, *P* < 0.001), and model R^2 increased from 0.322 to 0.374 (Supplemental Table S5).

**Table 5.** Assessment Coverage and Descriptive Summaries Across Available Early Repeated Assessments (RepeatKey 1-6)

RepeatKey	Assessments/subjects (n)	Coverage vs RK1 (%)	Mean status	Status SD	Any analgesics (%)	Any peaks (%)
1	80	100.0	51.34	25.26	77.5	41.2
2	77	96.3	53.79	21.9	71.4	45.5
3	70	87.5	56.86	23.29	57.1	48.6
4	64	80.0	61.5	23.3	42.2	35.9
5	52	65.0	62.48	23.05	42.3	44.2
6	45	56.3	61.71	24.7	48.9	51.1

*Note.* Each row summarizes all available assessments at that ordinal repeat index; later RepeatKey values therefore include fewer contributing subjects and do not require complete six-assessment follow-up. Within this early repeated-assessment subset, each subject contributed at most 1 observation per RepeatKey, so the assessment and subject counts were identical. Coverage percentages use RepeatKey 1 (n = 80) as the denominator. Structured reasons for non-completion were not prospectively recorded in this routine-care registry.

### Supplementary Results

Because pain peak intensity is conditionally relevant when pain peaks occur, missingness in peak intensity is expected when “no peaks” are reported. Accordingly, valid peak intensity responses were rare when no peaks were reported (12.1%) but were highly complete when peaks were present (86.5-94.1% across frequency categories) (Table S1). This reflects structural non-applicability of the conditional peak-intensity item rather than proof that these assessments were completely pain-free overall, because global status and wound pain were captured separately.

At the assessment level, however, primary-model completeness was limited: only 209/1627 status assessments (12.8%) met complete-case criteria. Complete assessments were concentrated in RepeatKey 1-6 (187/937, 20.0%) and were uncommon beyond RepeatKey 6 (22/690, 3.2%) (Table S10B). Compared with incomplete status assessments, complete assessments had lower concurrent status (median 60 [IQR 40-75] vs 70 [50-85]) and higher wound pain (median 3 [1-5] vs 2 [1-4]) (Table S10A), indicating that assessment-level missingness was unlikely to be random with respect to symptom burden. This pattern represents the main methodological constraint on inference in the present study.

In a simplified binary framework, overall status showed a clear gradient across the 4 combinations of any pain peaks (yes/no) and any analgesic use (yes/no) (Table S2; Figure S1). The highest mean status occurred when neither peaks nor analgesics were present (mean 70.7; median 75), whereas the lowest mean status occurred when both were present (mean 47.6; median 50) (Table S2). In a cluster-robust regression model, any peaks were associated with −14.45 points lower status (*P* = 7.36 × 10^-4^) and any analgesics with −18.15 points lower status (*P* = 2.23 × 10^-8^); the interaction term was not statistically significant (*P* = 0.10) (Table S3A). The estimated contrast between “any peaks & any analgesics” vs “no peaks & no analgesics” was −23.10 points (95% CI −29.80 to −16.39) (Table S3B).

As a missing-data robustness check, we refitted the mixed-effects model without peak intensity so that the larger dataset with status, peak frequency, and analgesic use could be retained (425 assessments from 135 subjects). In this broader model, more frequent peaks remained associated with worse status (3-5/day vs none: beta −13.37, 95% CI -20.35 to −6.38; >5/day vs none: beta −26.77, 95% CI -35.83 to −17.71), and both opioid-only (beta −10.29, 95% CI -19.43 to −1.15) and non-opioid-only use (beta −8.79, 95% CI -13.70 to −3.89) were associated with lower status relative to no analgesics (Table S11). Together with the peaks-present model, this broader sensitivity analysis supports the stability of the main construct-validity direction despite incomplete covariate capture.

Wound pain was generally low-to-moderate but widely distributed (median 2, IQR 1-4, range 0-10) (Table S4A). Wound pain correlated strongly with worse overall status (ρ = −0.473, *P* = 8.92 × 10^-90^, 1594 assessments) and correlated positively with pain peak intensity (ρ = 0.482, *P* = 1.23 × 10^-13^), pain peak frequency (ρ = 0.375, *P* = 1.60 × 10^-19^), and analgesic use (ρ = 0.323, *P* = 1.08 × 10^-11^) (Table S4B; Figure S2). In a joint model (202 complete assessments from 81 subjects), both pain peak intensity (β −4.19, *P* = 5.8 × 10^-5^) and wound pain (β −2.85, *P* = 1.34 × 10^-4^) were independently associated with worse status, and inclusion of wound pain increased explained variance (R^2^ 0.374 vs 0.322) while attenuating the peak-intensity coefficient by approximately 19% (Table S5). (Table S6A; Figure S3). In adjusted cluster-robust regression, status increased by + 1.49 points per +1 RepeatKey (*P* = 0.0417), while any peaks (−15.45, *P* = 4.26 × 10^-4^) and any analgesics (−17.53, *P* = 1.49 × 10^-7^) were each associated with worse status (Table S6B). In GEE logistic models, odds of any analgesic use decreased across RepeatKey (OR 0.74 per +1; *P* = 4.33 × 10^-7^), whereas odds of reporting any peaks did not change systematically (OR 1.00; *P* = 0.975) (Table S6C). Sex classification among subjects with status data was Male n = 127, Female n = 130 (Table S7A). In exploratory moderation analyses, there was no evidence that the pain peak intensity–status association differed by sex (interaction *P* > 0.6 in both known-sex and half-half sensitivity analyses), and age was not independently associated with status after adjustment (*P* = 0.63) (Table S7). Finally, restricting the mixed-effects model to assessments where peaks were present (168 assessments from 71 subjects) confirmed a robust negative association between peak intensity and status (β −4.78, *P* = 4.78 × 10^-8^) and showed that very frequent peaks (>5/day) were additionally associated with lower status compared with 1-2/day (β −17.02, *P* = 0.002) (Table S8).

## Discussion

### Filling the Postoperative “Black Box” With High-Frequency PROMs

Current spine outcomes research and routine postoperative care rely heavily on cross-sectional assessments conducted weeks to months after surgery. This traditional cadence fails to account for the immediate post-discharge period, which is characterized by rapid, nonlinear symptom changes. Recent mHealth and EMA studies show that infrequent sampling may miss important features of early recovery—a “black box” interval containing symptom patterns that may relate to later outcomes. In that context, our study does not introduce high-frequency monitoring as a novel concept and does not show that more frequent measurement is inherently superior for clinical decision-making.^8^ Rather, it asks a narrower question: whether a particularly brief, single-item global status item exhibits expected concurrent associations with pain-related constructs during this vulnerable window. Our intent is not to replace established instruments such as ODI or PROMIS, but to evaluate whether a low-burden summary signal can complement them in settings where repeated sampling is desired.^9,10^

### Construct Validity: Tracking Pain Peaks and Medication Use

The core validity question for a single, global high-frequency status item is whether it moves in the expected direction alongside clinically relevant concurrent constructs in early recovery. In our dataset, lower status scores were consistently associated with higher pain-peak intensity, more frequent pain peaks, greater analgesic use, and higher wound pain. These convergent patterns support construct validity for this specific use case. They do not establish that the item captures all relevant recovery domains, that it is interchangeable with established multidomain PROMs, or that it can by itself guide management decisions. Rather, the findings suggest that the score functions as a concise patient-reported summary signal of contemporaneous symptom burden within this routine-care pathway.^11^ High-frequency sampling may reduce reliance on broad retrospective summaries and can better situate symptom peaks in time,^12^ but the present study did not directly compare this item against diary-derived “gold standards” or established spine PROMs administered concurrently at the same frequency.

The dissociation visible in Figure 2 is therefore informative rather than contradictory. Mean status improved and analgesic use declined across repeated assessments, yet the proportion of assessments with any pain peaks remained relatively stable. Because the status item asked how patients felt overall today, it likely integrated several dimensions of recovery - not only breakthrough pain, but also background pain, function, sleep, confidence, and medication burden. Intermittent peaks may thus persist during mobilization even as overall recovery improves. This pattern supports interpreting the single-item status PROM as an integrative recovery signal rather than as a one-to-one surrogate for any single pain feature.

### Why “High-Frequency” Matters: Trajectories Over Time Points

A major value of high-frequency measurement is that it can describe recovery trajectories with greater temporal granularity.^6^ Across surgical fields, early postoperative pain trajectories separate into distinct subgroups that have been associated with later outcomes.^13^ Spine-specific research similarly shows that incomplete pain resolution in early recovery can accompany inferior later quality-of-life outcomes, and adult degenerative spine studies have linked early multidomain recovery patterns to longer-term outcomes and complication rates.^8^ Importantly, those prognostic observations come from prior literature rather than from the present analysis. Our study did not test long-term prediction or causal effects; it evaluated only whether the repeated global status item covaried with concurrent pain-related burden during early recovery.

### Alignment With Digital Monitoring and Implementation Transparency

Our findings extend a growing body of spine digital health literature moving toward ecological assessment. EMA studies demonstrate that brief, repeated symptom surveys are feasible and can correlate with established PROMs while capturing dynamics missed by retrospective instruments.^3^ Our study contributes by showing that an even briefer single-item global status measure remains linked to pain burden and analgesic use in routine postoperative monitoring. Postoperative remote monitoring platforms have also been shown to be feasible and acceptable, with some observational studies suggesting associations with reduced emergency utilization.^13^ Evidence further indicates that electronic pain data capture is congruent with conventional methods^5^ and that passive signals (eg, GPS-derived mobility) correlate with functional outcomes^14,15^. Within this landscape, the present measure may serve as a simple patient-reported summary signal that could be used alongside more detailed PROMs or objective streams. Accordingly, the present work is best interpreted as a construct-validation study embedded in routine care, supplemented by descriptive assessment-coverage metrics rather than by prespecified feasibility endpoints such as recruitment, refusal, or adjudicated dropout rates. A key limitation of the current validation scope is the absence of concurrent ODI or PROMIS measurements at the same repeated cadence. In our routine postoperative pathway, the high-frequency mobile workflow was intentionally kept brief to maximize adherence, whereas multidomain instruments were not collected every 2-3 days. As a result, the present study can address construct validity against concurrent pain-related measures, but not criterion/convergent validity against established multidomain PROM frameworks. This comparison is an important next step for future validation work.

### Robustness and Generalizability

A practical concern for any monitoring tool is whether its utility varies across demographic strata. Prior EMA work suggests that sex and age can influence symptom variability even when average severity is similar.^3^ However, in our exploratory models, the central construct-validity relationships—status tracking pain-peak burden and analgesic use—remained directionally consistent across age and sex. This robustness supports the interpretation that the measure may be broadly applicable rather than subgroup-specific in heterogeneous neurosurgical spine populations. At the same time, the present study evaluated construct validity, not implementation performance. Although lower status scores were consistently accompanied by worse pain and greater analgesic use, we did not test management algorithms, alert thresholds, or triage decisions. The most appropriate interpretation at this stage is therefore that the score provides a concise descriptive summary of early recovery burden, which could inform future implementation studies.

Although the present study was not designed to derive validated management thresholds, the observed cross-sectional distributions allow a cautious exploratory framework for future implementation studies. In descriptive terms, Global Status scores above 60 were more typical of lower-burden states (for example, no analgesics: mean 67.2; no pain peaks: median 60), scores in the 40-60 range reflected intermediate burden, and scores below 40 clustered with higher-burden states such as frequent pain peaks (>5/day: median 40, mean 34.6) and combined opioid plus non-opioid analgesic use (median 40, mean 41.9). Similarly, trajectories that remain below 40 across repeated early assessments or fail to show the expected upward trend over serial assessments may be reasonable candidates for additional clinical review in future prospective pathways. However, these proposed bands are hypothesis-generating only and were not derived against adjudicated complications, unplanned care use, or clinician-triggered interventions.

### Limitations and Future Directions

Our findings must be interpreted in light of several limitations. First, the analyses are observational and cannot establish causality; medication use may be both a response to pain and a marker of more severe postoperative states. Second, the primary outcome is a single-item global status measure. Although this low-burden format is practical for high-frequency assessment, it cannot disentangle specific recovery domains such as pain intensity, function, sleep, mood, or wound-related symptoms. Third, while longitudinal PROM collection is historically prone to high attrition, we implemented specific mitigation strategies to maximize data completeness, including seamless integration into the clinical electronic health record (EHR) workflow and active adherence monitoring by a dedicated study coordinator to resolve technical barriers. Nevertheless, high-frequency data remain susceptible to non-random missingness, as response likelihood may still vary with symptom severity. Fourth, pain peaks and medication use were self-reported and may be affected by subjective interpretation and short-term recall, although the frequent assessment schedule likely reduced the recall window compared with conventional follow-up. Fifth, no objective external validation measures, such as activity monitoring, medication dispensing data, or clinician-rated recovery markers, were available to triangulate the patient-reported measures. Finally, this was a single-center cohort of elective degenerative lumbar decompression cases at an academic tertiary center, which may limit generalizability to other practice settings, emergency presentations, non-degenerative indications, or more complex spinal procedures. Future work should examine whether repeated status trajectories predict subsequent clinical outcomes and whether combining this item with passive or multidomain measures improves recovery phenotyping. Within the early repeated-assessment summaries, available observations declined from 80 at RepeatKey 1 to 45 at RepeatKey 6 (56.3% relative retention). Because recruitment/refusal denominators and structured reasons for non-completion were not prospectively captured, we could describe coverage loss but could not distinguish technical barriers, clinical recovery, disengagement, or other causes of missingness. In observed subject-level comparisons, included and excluded subjects were similar in age, sex, earliest postoperative status, and earliest wound pain, but complete-case assessments were concentrated earlier in follow-up and in more symptomatic observations. Missingness therefore appears to reflect a mixture of structural mechanisms and informative non-response. We did not perform formal multiple imputation because peak-intensity values were partly structurally undefined when no peaks were reported.

## Conclusion

In summary, our high-frequency status PROM offers a scalable method to characterize recovery during the post-discharge “black box.” The tool demonstrated strong construct validity by closely tracking pain-peak burden and analgesic use, with findings that were directionally consistent across demographic factors. These results support further evaluation of high-frequency PROM monitoring as a pragmatic approach to describe postoperative recovery trajectories; prospective studies should determine whether such monitoring improves clinical decision-making, resource use, or patient outcomes.

## Supplemental Material

Supplemental Material - High-Frequency Global Postoperative Status PROMs Track Pain Peaks and Analgesic Use After Degenerative Lumbar Spine Surgery Supplemental Material for High-Frequency Global Postoperative Status PROMs Track Pain Peaks and Analgesic Use After Degenerative Lumbar Spine Surgery by Pavlina Lenga, Robin Fleige, Max Christian Blumenstock, Matthias Ganzinger, Sebastian Ille, Sandro M. Krieg and Martin Dugas in Global Spine Journal

## Data Availability

The datasets generated during and/or analyzed during the current study are available from the corresponding author on reasonable request.*

## Notes

1. Overall postoperative status (1-100): Patient-reported global health status today after spinal surgery; higher scores indicate better status.

2. Pain peak frequency (categorical): None; 1-2/day; 3-5/day; >5/day.

3. Pain peak intensity (0-10): If pain peaks occur: maximum intensity on a 0-10 scale.

4. Analgesic use (categorical): No analgesics; opioid analgesics; non-opioid analgesics; opioid + non-opioid.

5. Wound pain (0-10): Current wound pain intensity on a 0-10 scale.

6. Repeat assessment index (RepeatKey): Ordinal index of repeated assessments; not necessarily equal time intervals.

7. Age (years): Derived from the dataset age field and summarized per subject (median across available records).

8. Sex: Inferred from completion of sex-specific form versions (female vs male).
