# Supplementary material for: High-Frequency Global Postoperative Status PROMs Track Pain Peaks and Analgesic Use After Degenerative Lumbar Spine Surgery
Source: Global Spine J. 2026 Jun 5:21925682261449153. Online ahead of print. doi: 10.1177/21925682261449153 (PMC13241382; doi:10.1177/21925682261449153)
Supplement: Supplemental Material - High-Frequency Global Postoperative Status PROMs Track Pain Peaks and Analgesic Use After Degenerative Lumbar Spine Surgery [file sj-pdf-1-gsj-10.1177_21925682261449153.pdf]

Supplementary material

Supplementary Methods

MyEDC is a browser-based HTML5 application accessible on smartphones, tablets, and computers without local installation. In the institutional setup, an EDC proxy located in a demilitarized zone with layered firewall protection mediated external access. A secure Java/Spring job service dispatched personalized, single-use email links, and only authenticated questionnaire submissions were committed to the internal database. Submitted forms were automatically rendered as PDF documents and integrated into the hospital information system (i.s.h.med; Oracle Cerner). Within the routine postoperative workflow, invitations were generated on an approximately every-2-to-3-day cadence for up to 6 weeks. Exact submission timestamps were retained at the platform level, but the present analyses operationalized follow-up position using the ordinal RepeatKey variable rather than exact elapsed days.

Table S1. Skip logic / missingness: valid pain peak intensity responses by pain peak frequency category.

Pain peak frequency	n_with_freq	n_with_intensity_valid	pct_intensity_valid
None	356	43	12.1
1–2/day	111	96	86.5
3–5/day	50	45	90
>5/day	34	32	94.1

Table S2. Overall status by combined presence of pain peaks and analgesic use (binary 2×2 groups).

Group	Assessments (n)	Subjects (n)	Mean	SD	Median	Q1	Q3	% ≥80
Any peaks / Any analgesics	142	61	47.63	23.97	50	30	65.75	11.3
Any peaks / No analgesics	46	33	56.28	25.36	62.5	40	75.75	21.7

No peaks / Any analgesics	100	62	52.58	22.17	57	40	65	13.0
No peaks / No analgesics	137	72	70.73	20.5	75	60	90	46.0

Table S3A. Cluster-robust regression: association of any peaks / any analgesics with overall status.

Term	Coef	SE	p-value	CI low	CI high
Any peaks (vs none)	-14.447	4.28	0.000736	-22.836	-6.059
Any analgesics (vs none)	-18.15	3.245	2.23e-08	-24.51	-11.79
Interaction (peaks × analgesics)	9.501	5.795	0.101	-1.857	20.859

Table S3B. Contrast: (any peaks & any analgesics) vs (no peaks & no analgesics).

Contrast	Coef	SE	CI low	CI high
(Any peaks & Any analgesics) − (No peaks & No analgesics)	-23.096	3.42	-29.799	-16.393

Figure S1. Overall status by combined presence of peaks and analgesics (2×2).

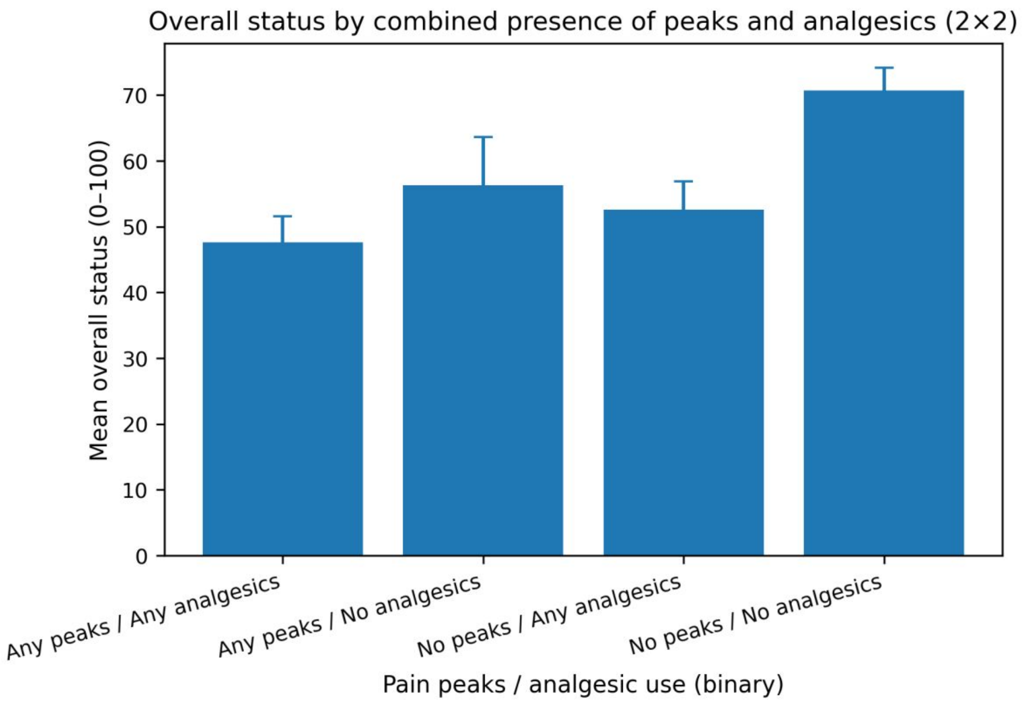

Table S4A. Wound pain (0–10): overall distribution.

Assessments (n)	Subjects (n)	Mean	SD	Median	Q1	Q3	Min	Max
1604	255	2.65	2.38	2	1	4	0	10

Table S4B. Correlations of wound pain with overall status and pain peak variables.

Pair	Method	Correlation	p-value	Assessments (n)	Subjects (n)
Wound pain vs Overall status	Spearman	-0.473	8.92e-90	1594	255
Wound pain vs Pain peak intensity	Spearman	0.482	1.23e-13	210	86
Wound pain vs Pain peak frequency	Spearman	0.375	1.6e-19	540	155
Wound pain vs Analgesic use	Spearman	0.323	1.08e-11	422	134

Note: Extremely small p-values in this table were re-checked against the original R output and are reported in scientific notation.

Table S5A. Joint model metadata (status outcome).

Assessments (n)	Subjects (n)	R^2 (with wound pain)	R^2 (without wound pain)	Peak intensity coef (without wound pain)	Peak intensity coef (with wound pain)	Attenuation of intensity effect (%)
202	81	0.374	0.322	-5.17	-4.19	19.0

Table S5B. Joint model key coefficients (status outcome).

Term	Coef	SE	p-value	CI low	CI high
Pain peak intensity (0–10)	-4.188	1.041	5.77e-05	-6.229	-2.147
Wound pain (0–10)	-2.853	0.747	0.000134	-4.318	-1.389

Figure S2. Wound pain relationships.

Panel A: wound pain vs pain peak intensity. Panel B: wound pain by peak frequency. Panel C: overall status vs wound pain.

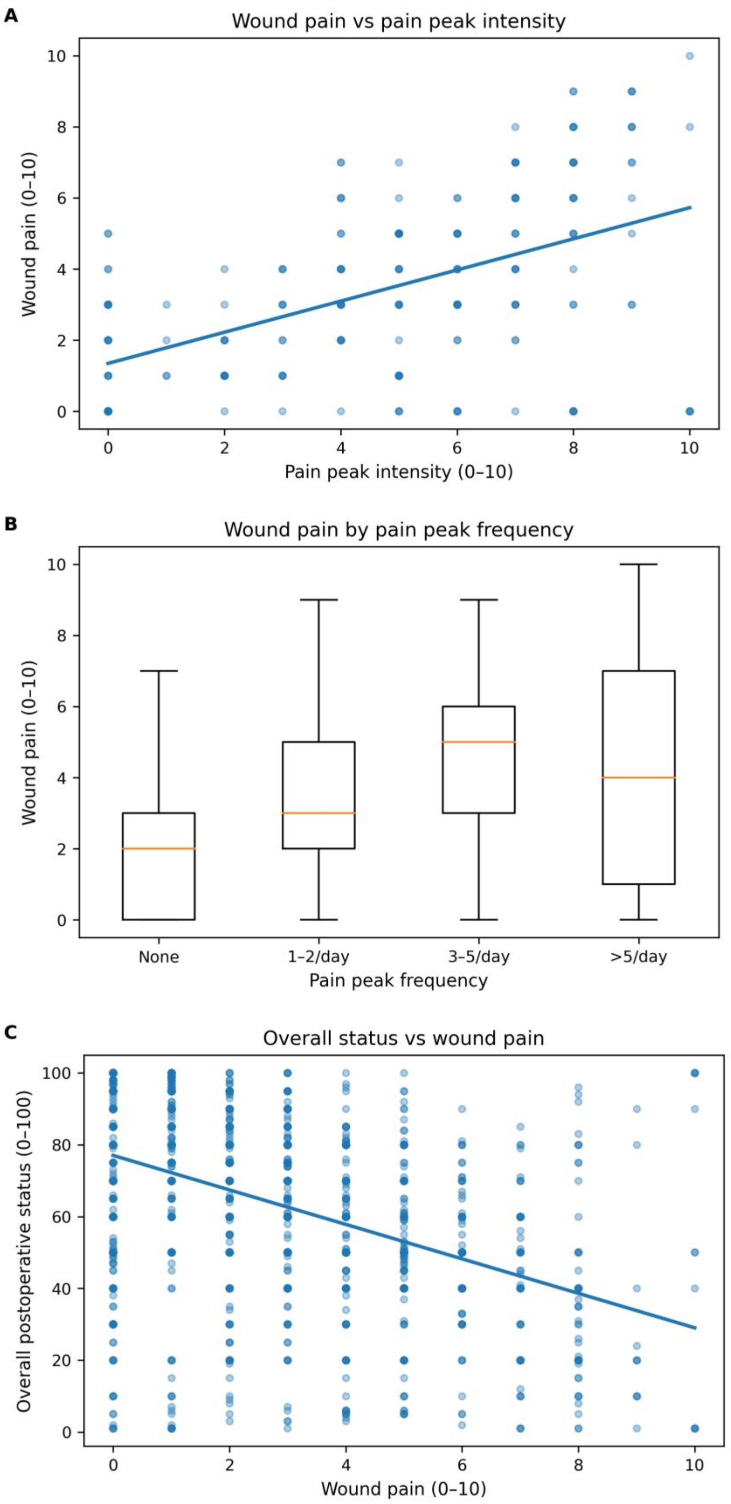

Table S6B. Cluster-robust regression: overall status vs timepoint, adjusted for any peaks and any analgesics.

Term	Coef	SE	p-value	CI low	CI high
Timepoint index (per +1)	1.486	0.73	0.0417	0.056	2.917
Any peaks (yes vs no)	-15.445	4.384	0.000426	-24.037	-6.854
Any analgesics (yes vs no)	-17.529	3.336	1.49e-07	-24.068	-10.99
Interaction (peaks × analgesics)	10.745	5.902	0.0686	-0.822	22.312

Table S6C. GEE logistic models: change in any peaks / any analgesics across timepoints (OR per +1 timepoint).

Outcome	OR per +1 timepoint	CI low	CI high	p-value
Any analgesics (binary)	0.743	0.663	0.834	4.33e-07
Any peaks (binary)	1	0.89	1.128	0.975

Table S7A. Sex interaction model (known sex only): overall status ~ pain peak intensity × sex (adjusted).

Term	Coef	SE	p-value	CI low	CI high
Pain peak intensity (per +1)	-4.441	1.487	0.00283	-7.356	-1.526
Male (vs Female)	3.852	10.037	0.701	-15.819	23.524
Interaction: intensity × Male	-0.316	1.641	0.847	-3.533	2.9

Table S7D. Sensitivity analysis (unknown sex split 50/50): overall status ~ pain peak intensity × sex (adjusted).

Term	Coef	SE	p-value	CI low	CI high
Pain peak intensity (per +1)	-4.5	1.178	0.000133	-6.808	-2.191
Male (vs Female) [half-half unknowns]	6.205	8.518	0.466	-10.491	22.901
Interaction: intensity × Male [half-half]	-0.575	1.416	0.685	-3.349	2.2

Table S7F. Age-adjusted model: overall status ~ pain peak intensity + age (adjusted).

Term	Coef	SE	p-value	CI low	CI high
Pain peak intensity (per +1)	-4.357	0.971	7.19e-06	-6.261	-2.454
Age (years, per +1)	-0.088	0.181	0.627	-0.443	0.267

Figure S4. Overall status vs pain peak intensity stratified by sex

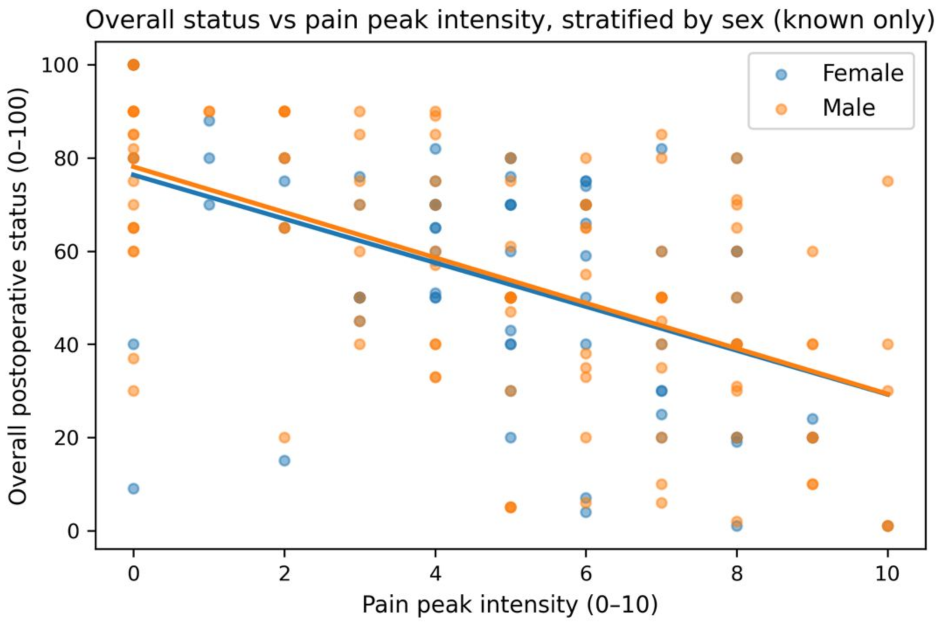

Table S8A. Peaks-only mixed model metadata (restricted to assessments with pain peaks present).

Model	Outcome	Dataset	Reference frequency category	Assessments (n)	Subjects (n)	ICC (random intercept)
Mixed-effects linear model (random intercept per subject)	Overall postoperative status (1–100)	Restricted to assessments with pain peaks present (frequency > None)	1–2/day	168	71	0.309

Table S8B. Peaks-only mixed-effects model estimates.

Term	Coef	SE	CI low	CI high	p-value
Intercept	79.938	5.083	69.976	89.9	9.77e-56
Pain peak frequency: 3–5/day vs 1–2/day	-6.63	3.943	-14.357	1.098	0.0927
Pain peak frequency: >5/day vs 1–2/day	-17.019	5.51	-27.818	-6.22	0.00201
Analgesics: opioid vs none	1.337	6.421	-11.248	13.921	0.835

Analgesics: non-opioid vs none	3.366	4.122	-4.713	11.444	0.414
Analgesics: opioid + non-opioid vs none	11.576	7.623	-3.364	26.517	0.129
Pain peak intensity (per +1 on 0–10 scale)	-4.78	0.876	-6.496	-3.064	4.78e-08

Table S9. Observed subject-level characteristics: included vs excluded from the primary complete-case mixed-effects model.

Characteristic	Included (n=82)	Excluded (n=175)	p-value
Age, years (median [IQR])	63 [56-72]	66 [54-75]	0.710
Male sex, n (%)	42 (51.2%)	84 (48.0%)	0.630
Earliest postoperative Global Status, median [IQR]	60 [40-70]	50 [40-70]	0.638
Earliest wound pain, median [IQR]	4 [2-5]	4 [2-5]	0.929
Valid Global Status assessments per subject, median [IQR]	5 [3-6]	4 [2-9]	0.568

Note: Age was available in 68/82 included subjects and 151/175 excluded subjects. Earliest wound-pain data were available in 81/82 and 174/175 subjects, respectively. P-values are exploratory and based on observed-case comparisons.

Table S10A. Assessment-level characteristics of complete vs incomplete status assessments.

Metric	Complete (n=209)	Incomplete (n=1418)	p-value
Assessments (n)	209	1418	
Subjects (n)	82	241	
Concurrent Global Status, median [IQR]	60 [40-75]	70 [50-85]	<0.001
Concurrent wound pain, median [IQR]	3 [1-5]	2 [1-4]	<0.001

Note: Status assessments were classified as complete when Global Status, pain peak frequency, pain peak intensity, and analgesic category were all simultaneously available and within range.

Table S10B. RepeatKey-specific availability of complete-case data among status assessments.

Assessment stratum	Status assessments (n)	Complete assessments (n)	Availability
All status assessments	1627	209	12.8%
RepeatKey 1	213	32	15.0%
RepeatKey 2	193	36	18.7%
RepeatKey 3	167	33	19.8%
RepeatKey 4	146	28	19.2%
RepeatKey 5	119	28	23.5%
RepeatKey 6	99	30	30.3%
RepeatKey >6	690	22	3.2%

Table S11A. Missing-data robustness model metadata (broader mixed-effects model without peak intensity).

Field	Value
Model	Mixed-effects linear model (random intercept per subject)

Outcome	Overall postoperative status (1-100)
Dataset	Status + pain peak frequency + analgesics (no peak-intensity requirement)
Assessments (n)	425
Subjects (n)	135
ICC (random intercept)	0.308

Table S11B. Missing-data robustness mixed-effects model estimates (status outcome; no peak-intensity requirement).

Term	Coef	SE	CI low	CI high	p-value
Intercept	66.043	2.074	61.979	70.107	<0.001
Pain peak frequency: 1-2/day vs None	-2.771	2.634	-7.934	2.393	0.293
Pain peak frequency: 3-5/day vs None	-13.367	3.565	-20.353	-6.380	<0.001
Pain peak frequency: >5/day vs None	-26.773	4.622	-35.831	-17.714	<0.001
Analgesics: opioid vs none	-10.290	4.665	-19.434	-1.146	0.027
Analgesics: non-opioid vs none	-8.794	2.502	-13.697	-3.890	<0.001
Analgesics: opioid + non-	-10.567	6.533	-23.373	2.239	0.106

opioid vs none					

Reference categories: pain peak frequency = None; analgesic use = no analgesics.

Table S12. Available baseline sample characterization from the questionnaire-centered export.

Field	Value
Sex, n (%)	Female 130/257 (50.6%); Male 127/257 (49.4%)
Age, median [IQR], years	63 [54-72]
Height, mean +/- SD, cm (n=190)	178.4 +/- 6.5
Weight, mean +/- SD, kg (n=190)	90.5 +/- 16.2
BMI, mean +/- SD, kg/m^2 (n=190)	28.4 +/- 4.7
Baseline disability summary from 10-item disability block, median [IQR], % (n=184)	46 [33.3-60]
Baseline pain-intensity item from same block, median [IQR], 0-5 (n=189)	3 [2-4]

Note: This descriptive table summarizes only fields that were retained in analyzable form in the questionnaire-centered export. The disability summary was derived exploratorily from the structured 10-item baseline disability block by recoding 1-6 responses to 0-5 and averaging across available items when at least 8/10 items were present. A standalone harmonized preoperative pain NRS, reliable per-subject LDH-vs-SCS subgroup counts, structured comorbidity coding, and discharge disposition were not uniformly retained and therefore could not be tabulated systematically.
